# The interindustry wage differentials by sector in China: What is the role of union density?

**DOI:** 10.3389/fsoc.2022.949293

**Published:** 2022-09-23

**Authors:** Mingming Li

**Affiliations:** Department of Economics and Business, Central European University, Vienna, Austria

**Keywords:** interindustry wage differential, union density, administrative monopoly, two-stage approach, instrumental variable

## Abstract

Although how union density affects interindustry wage differentials has long been discussed, there is a paucity of empirical research relevant to China. The trade-union system in China has been criticized for a long time because the Chinese Communist Party can influence union density and indirectly affect interindustry wage differentials through non-market mechanisms, such as administrative monopolies. This study explores the impact of union density on interindustry wage differentials in the context of administrative monopolies. The research takes a two-stage estimation approach after scrupulously integrating and conforming more than 40,000 individual data from the Urban Household Survey and various yearbooks from years 2004, 2008, and 2013. In the first stage, the individual wages are regressed with industry-sector dummies to obtain the wage-differential coefficients. Furthermore, union density is considered as a core variable to create regressions to the interindustry wage differential coefficients obtained in the first stage using administrative monopolies and labor safeguards as instrumental variables. It is found that although the union density was expected to increase wage differentials in industries, its influence diminished in 3 years under study. Administrative monopolies can indirectly affect wage differentials through union density. The support to grassroots unions in non-administrative monopolies industries and the opening up of industry to the private sector will help to overcome this dilemma.

## Introduction

Wage inequality increased alongside the rapid economic growth experienced in China in recent decades. Researchers attempt to explain it from different perspectives, such as the rural–urban transition (Sicular et al., 2008; Ma, 2018a; Wang et al., 2019), returns on education (Zhu and Lou, 2011; Zhou, 2014), sector (Ma, 2018b; Cooke, 2020), and gender (Appleton et al., 2014; Ge and Yang, 2014; Iwasaki and Ma, 2020). However, there is relatively scant research focusing on the issue of interindustry wage inequality, which is increasing in many countries according to Carruth et al. (2004) and Abowd et al. (1999).

Interindustry wage differentials can be caused by various factors, such as human capital (Bridges, 2018; Kimura et al., 2022), globalization (Chen et al., 2010), urban–rural differences (Chen et al., 2010; Wang et al., 2018), and union activity (Dickens and Katz, 1987; Krueger and Summers, 1988; Mokre and Rehm, 2020). Although unions can influence interindustry wage differentials, not alike western countries, unions are always influenced by administrative monopolies in China. The concept of the administrative monopoly is defined as:

*monopolistic behavior that is supported by government or regulatory agencies at both central and regional levels. The central government protects specific sectors or industries through exercising administrative authority. Local government exerts administrative power over enterprises within the region and protect the profits of these enterprises by creating market barriers (Ma*, *2020**)*.

Therefore, in order to explore the relations between union and wage differentials, it is necessary to explain how administrative monopolies influence unions. This study begins with the relation between administrative monopolies and unions.

**Figure 1:**
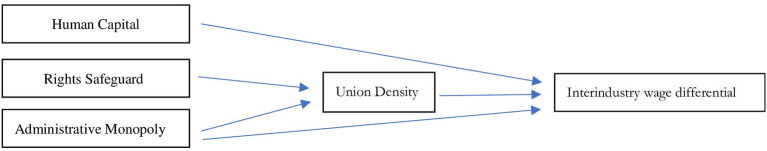
The expected relationship between union density and wage.

Owing to the deep influence of the Soviet Union and socialist ideology, the proportion of publicly owned capital in certain industries and sectors in China (especially naturally monopolistic industries, such as energy, finance, electricity, and other infrastructures) is much higher than in other industries, with strong government control. Usually, the union density in these industries and sectors is much higher than in others. In the Chinese context, administrative monopolies not only influence the wage differentials directly, but also do so through indirectly, through trade unions. Furthermore, union density can also be influenced by other factors, such as labor safeguards. A framework of how union density can influence interindustry wage differentials s presented in Figure 1.

The article investigates solutions for three core questions: 1. How does union density influence interindustry wage differentials? 2. How do administrative monopolies influence interindustry wage differentials directly, or indirectly, through union density? 3. How do other factors, such as human capital and labor, safeguard interindustry wage differentials?

Compared to the previous literature, this paper offers several main contributions. Firstly, as there are no meso data on industry wage levels, this study uses a two-stage approach to estimate the interindustry wage differentials through micro data, which is used less often in the Chinese literature. Second, the trade unions in China are influenced by the Chinese Communist Party (CCP). If the effect of trade-union density on the interindustry wage differentials is considered alone, the estimates are bound to be biased. To address this issue, this study regresses union density using administrative-monopoly indicators, such as public-sector share and the number of people in the labor forces of state-owned enterprises, as instrumental variables (i.e., the influence of the Communist Party is included in union density), and then regresses the interindustry wage differentials again to obtain the effect of union density. Third, traditional articles only analyse unions by either industry or sector, and not consider both. This paper considers industry and sector as a single dummy variable (i.e., a cross-section of industry by sector). Finally, this paper is the most recent study of labor-force and union data in China. We use cross-sectional data from 2004, 2008, to 2013 which can be found in the Chinese Trade Unions Statistics Yearbook, which has not been updated since 2013. The more recent China Labor Statistical Yearbook no longer includes industry-specific trade-union information. In short, this paper is the most recent summary of the study of the effect of union density on interindustry labor wage differentials under administrative-monopoly conditions in China.

This study uses a two-stage approach to examine interindustry wage differentials. This two-stage method was pioneered by Krueger and Summers (1988) and Winter-Ebmer (1994). In the first stage, individual wages are regressed on individual and worker-specific characteristics and industry-sector dummies to yield the industry-sector wage premium. In the second stage, the adjusted industry-sector premium is regressed on cross-section data with core-variable union density, using administrative monopolies as instrument variables.

The rest of this article is arranged as follows. Section Literature Review is a review of the literature on union and wage differentials. Section History of Chinese Union Development introduces the development of unions in recent years, and Section Methodology describes the two-stage regression method. Section Data presents the data source and a descriptive explanation, and Section Empirical Analysis explain the results of the empirical analysis. Finally, Section Conclusion and Policy Suggestions summarizes the study and offers policy recommendations.

## Literature review

From a theoretical perspective, three main theories can explain wage differentials. The first theory involves the normal functioning of competitive labor markets, discussing compensating differential levels among workers. The model developed by Lucas (1988) is regarded as the starting point in the examination of the impact of human capital on wages, which was empirically investigated by Krueger and Summers (1988), focusing on individual characteristics. The second theory involves institutional factors, such as the ability of unions to affect wages. The third is the efficiency wage theory, which shows that employers try to increase profits by paying workers above-market wages (Dickens and Katz, 1987; Goux and Maurin, 1999).

Early studies used average industry wages to measure wage differentials across industries. This approach treats industry wages as independent of workers' characteristics (Goh and Javorcik, 2007). However, worker and firm heterogeneity could become an obstacle, which can account for 90% in France (Abowd et al., 1999). More recent studies mainly measured wage variations across industries after controlling for worker and firm effects.

From an empirical perspective, the relationship between unions and labor wages is discussed extensively in the related literature (Card et al., 2017), and there is no highly unified opinion about how and in which direction unions influence wage differentials.

Before the 1980s, most economists agreed that unions tend to increase overall inequality by creating an average payment gap between union and nonunion sectors (Johnson, 1975). According to most studies in the US (Lewis, 1983; Bryson, 2014; Zhang, 2019), this gap can reach ~10–30%. Moreover, the effects on different sectors and industries are not uniform (Jaumotte and Osorio, 2015; Macpherson and Hirsch, 2021).

However, the widening of the wage gap between unionized and nonunionised industries and sectors does not necessarily mean that unions increase the overall degree of wage dispersion (Freeman and Medoff, 1984; Krueger and Summers, 1988; Wang and Lien, 2018). Freeman (1980) argued that union wage setting tends to reduce wage dispersion between skilled and less-skilled or low-wage industry workers and between high- and low-paying establishments, thereby leading to a ‘within-sector’ inequality effect that may offset the ‘between sector’ effect arising from the average union wage gap. In comparative studies using OECD countries as samples, Blau and Kahn (1996) and Kahn (2000) suggested the negative correlation between union density and wage inequality.

From the 1970s, researchers started to focus on union density and had used the decline in union density in public organizations in Britain and other countries to explain the increase in wage inequality in these countries (Card, 2001; Fortin et al., 2021). It was found that the decrease in union density could explain 15–20% of the wage inequality. Scruggs and Lange (2002) pointed out that the decline in union density mainly arises from changes in the extent of globalization, institutions, and industries. Fitzenberger et al. (2013) emphasized that researchers should distinguish between union density, firm-level coverage, and individual-level coverage, with higher union densities enhancing the effect of coverage. Barth et al. (2020) focused on changes in the tax subsidies for union members in Norway to determine the effect of changes in union density at the firm level on productivity and wages, and found that increasing union density at the firm level resulted in large increases in productivity and wages. Therefore, we have Hypothesis 1:

*Hypothesis 1: Union density influences interindustry wage differentials*.

The literature on whether Chinese unions can help reduce wage inequality is limited, and the union-wage-effect results are inconclusive. Budd et al. (2014) used provincial data from 1994–2008 to show that union density does not affect average wages but is positively related to total productivity and output. From the perspective of the dual identity of Chinese unions in public and workers' organizations in a market economy, Chen (2003) believed that role conflicts limit the practical effects of Chinese unions. Since unions fail to perform their functions, over half of workers believe that unions are insignificant, including their role in wage distribution. Several studies found a positive and statistically significant union effect on labor productivity, but not on profitability (Lu et al., 2010).

However, other studies presented different opinions. Extensive microdata samples were used in 2008 to determine whether Chinese unions bring increase staff wages. The results showed that compared with nonunion workers, union workers have significantly higher wage levels and experience lower wage inequality (Jain-Chandra et al., 2018). Yao and Zhong (2013) showed that the presence of unions is significantly associated with high wage rates and pension coverage for workers, as well as a wide range of welfare indicators.

Sheng et al. (2015) and Yu and Zhang (2013) argued that administrative power plays an important, and even decisive role in access, restriction, and pricing in many important industrial fields in China. The enterprises keep all the profit due to the existence of administrative monopolies. For example, Dai et al. (2019) and Huang (2016), taking the Chinese aviation industry as an example, estimated the net loss in social welfare, the increase in total costs, and the transfer effect of welfare brought about by administrative monopolies. Yan and Pu (2014) evaluated the forms and origins of administrative monopolies in the petrochemical industry and argued that administrative monopolies cause factor-price distortions. Li (2010) and Xu et al. (2021) reached a similar conclusion from an analysis of China's toll road and taxi industries. Most of these monopoly industries, such as oil and highways, belong to the public sector and are operated by large state-owned enterprises in China (i.e., Sinopec Group). This means that administrative monopolies can distort the distribution of economic factors and use price discrimination to create interindustry. wage differentials. At the same time, as mentioned in the introduction, the organization of laborers by industry sector (i.e., those under the influence of administrative monopolies) tends to affect union density. Consequently, it is necessary to consider sectors and industries comprehensively. Therefore, this study proposes Hypothesis 2 and Hypothesis 3:

*Hypothesis 2: The administrative monopoly can influence the interindustry wage differentials directly by sector*.

*Hypothesis 3: The administrative monopoly can also influence the interindustry wage differentials through union density indirectly, by sector*.

In addition to union density, other factors also affect interindustry wage differentials. A typical example is human capital (Weinberg, 2001; Sullivan, 2010). It is well known that, as Mincer (1974) pointed out, schooling and professional experience have a direct impact on individual earnings, according to human capital theory. Academics have also examined the effects of education and professional experience on industry wage differentials in recent years (Sullivan, 2010; Firpo et al., 2018; Valletta, 2018), as these two human-capital variables are the main factors influencing the choice of sectors and subsequent wage differentials.

*Hypothesis 4: Human capital can influence interindustry wage differentials*.

## History of Chinese union development

In the early stage after the foundation of the country (1949–1978), monopoly industries represented the goals of the nation and were crucial to economic growth, and the CCP, which remains the ruling party in China, claimed to be a working-class party. Therefore, union density is naturally high in certain industries due to history and ideology. In other words, the union density in industries with a large ratio of public employees and capital is naturally higher than that in other industries. Moreover, publicly owned organizations typically pay higher wages than those in other sectors (the private sector or collective economy), as well as offering status and social-welfare advantages. In this period, China's overall economic development was low, and the wage gap between industries was small; however, this changed as economic growth and inequality increased after 1978. Thus, the role of unions in interindustry wage differentials under the influence of the administrative monopoly must be carefully discussed.

The reform and opening up of China's economy began in 1978 and underwent a series of market-structure transformations and industrialization adjustments. Since the 1990s, the proportion of the private sector has increased significantly, and its status has risen rapidly. The resulting structure influenced rapid economic development, social welfare, and wage differentials. With the booming of the private sector and the birth of thousands of small and medium-sized enterprises, the All-China Federation of Unions requested the establishment of union organizations in the private sector in 1995 and their promotion in 1998.

**Figure 2:**
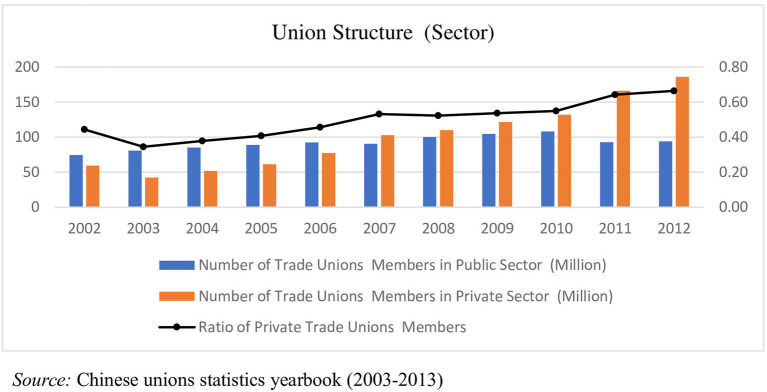
Union members structure by sector.

**Figure 3:**
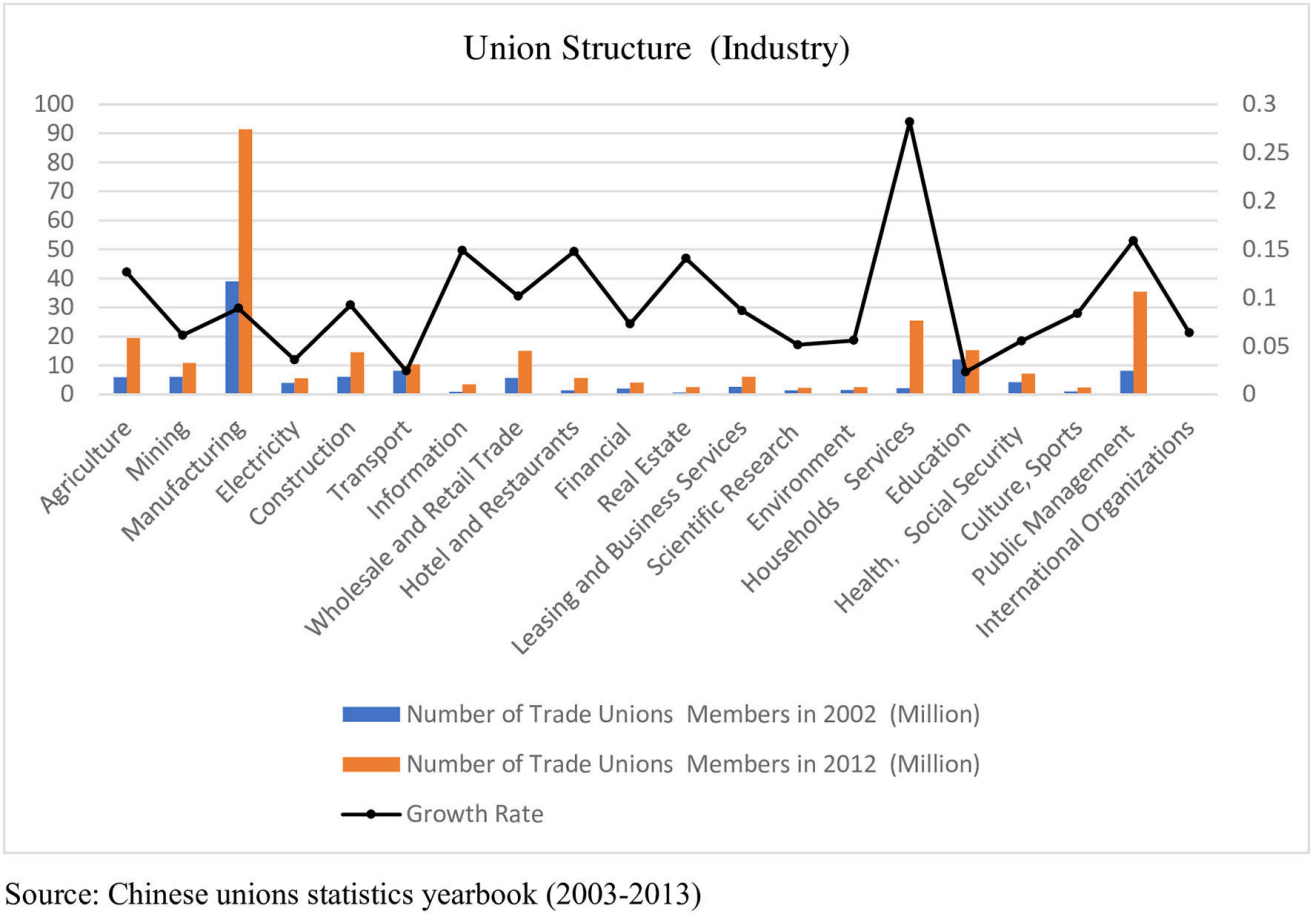
Union members structure by industry.

Overall, unions in China experienced an explosive growth after the 21st century, as shown in Figure 2. In 2002, the number of unions in China did not exceed 150 million, but in 2012, it exceeded 280 million. At the same time, the composition of Chinese labor unions underwent tremendous changes. In terms of the gender structure, the proportion of female members increased from 35% in 2002 to 38% in 2012. During this period, the number of employed women increased from 50 million to more than 100 million, due to the development of the market economy. In 2002, many state-owned enterprises were yet to be reformed or marketised from a sectoral perspective; thus, private-sector union members accounted for ~44%. With the development of multiple sector systems, union members in the private sector accounted for two thirds of the total by 2012. In terms of the industrial structure, the manufacturing industry was undoubtedly the largest, followed by the public-management and residential-services industries. Among these industries, public management demonstrated the fastest growth rate, and the manufacturing industry had the largest absolute number of members (Figure 3). At the same time, the number of disputes related to labor wages also started to increase, reflecting one point from the side. The China Labor Statistics Yearbook recorded 380,751 disputes related to labor wages in 2018, accounting for ~40% of all cases, compared with only 45,172 in 2001 (Chi et al., 2019).

The unions in China differ in the traditional sense from those in Europe and the United States. Union coordination typically involves the following steps. Firstly, workers present their problems to the union. Secondly, the union communicates with the enterprise or high-level government bureaus and finally negotiates through consultations. Chinese labor unions infrequently fight for their rights through strikes (though this practice is theoretically allowed). On one hand, a strike entails very high economic costs. On the other hand, a strike requires government approval and faces political issues (Chen, 2010; Chan and Hui, 2012). Most importantly, unions in China operate independently and seek solutions to actual problems in most cases.

In this study, we do not intend to discuss the nature of Chinese unions. Instead, we treat them as institutions in a unique economic environment, since unions in China are mainly self-managed undertaking all measures independently, although they remain politically controlled by the CCP. Unions comprise various levels and follow hierarchical systems. In addition, Chinese union leaders are generally selected and appointed by a party organization from a group of organizations to which the union belongs. Union leaders typically have certain abilities and prestige and enjoy satisfactory relationships with workers and the CCP. Their roles mainly include helping the CCP stabilize society and coordinate income conflicts between capitalists and workers. Other union functions involve assisting in employment issues and fighting for increased social welfare. In addition to this interpretation of Chinese unions, we use the administrative-monopoly index to conduct a regression on union density to include the influence on wage differentials. In other words, the impact of the CCP on union density is fully considered in the analysis of the effect of union density on wage differentials.

## Methodology

This research uses a two-stage model (Krueger and Summers, 1988; Ebmer, 1990; Winter-Ebmer, 1994) for the analysis. The advantage of this research method is that the topic can be examined at the industry-sector level by controlling individual human capital and other factors. Taking advantage of the influence of industry-sector characteristic factors on labor wages can eliminate the disturbing effect of individual-level factors in the second stage. In the first-stage regression, microsamples were used, mainly to control for individual factors affecting labor wages to obtain the industry wage differential coefficients. In the second-stage regression, based on the wage-differential coefficient estimated in first stage, the corresponding characteristic industry variables (e.g., union density) were used for the regression, and we determined how these characteristic industry variables influence wage differentials.

The classification method is a crucial prerequisite for ensuring the consistency of the coefficients. As most studies use only one-digit industry classification (using around 10 to 30 industries), highly aggregated industry categories can cause the absence of sufficient data points in second-stage regressions. To solve this problem, this study adopted an industry-sector dummy variable to improve the industry-classification degree used, as in Ebmer (1990) and Winter-Ebmer (1994). The setting matches the nature of the industry, which is strongly linked to sector in the Chinese labor market, as mentioned at the beginning of this article. Through this detailed classification method, the influence of sectors on individual industries can be tested. Therefore, based on the income function developed by Mincer (1974), in connection with the industry-sector characteristics and other control variables, the two-stage mode can be established, and the first stage is as follows:


(1) $$\begin{array}{l} \ln\;w_{ijt}\;=\;\alpha_{0}\;+\;\alpha_{jk}Z_{ijt}I_{ikt}\;+\;X_{it}\theta \;+\;\varepsilon_{it} \end{array}$$

where ln *w*_*ijt*_ represents log yearly wages for individual *i* working in industry *j* in year *t*. *Z*_*ijt*_ are dummies for industry affiliation *j* and ownership type *k*, *X*_*it*_ is a vector of worker-specific characteristics, which include human capital (education, working experience), individual feature (gender, marital status), occupations, and districts, and ε_*it*_ is the error term. In the first stage, α_*jk*_ is the industry wage differential coefficient by sector.

The estimated wage differential coefficient α_*jk*_ should be normalized to show the proportional difference in wages between an employee in each industry and an average employee.


(2) $$\begin{array}{lll} \tau_{jk}\; & = & \;\alpha_{jk}-\bar{WA} \end{array}$$

(3) $$\begin{array}{lll} \bar{WA}\; & = & {\text{Σ}}_{jk}\alpha_{jk}\;n_{jk}/Sigma_{jk}n_{jk} \end{array}$$

where τ_*jk*_ is the adjusted wage differential coefficient, $\bar{WA}$ is the employment-weighted average wage premium, and *n*_*jk*_ is the number of employees in industry *j* and sector *k*.

Thus, τ_*jk*_ can be applied in the second-stage regression, as follows:


(4) $$\begin{array}{lll} \tau_{jk}\; & = & \;c\;+\;\lambda U_{jk}\;+\;\gamma AH_{jk}\;+\text{ δ}AX_{jk}\;+\;\varepsilon_{jk}, \end{array}$$

where *U*_*jk*_ is the union density in a specific industry by sector. In addition to focusing on the core variable, namely, union density, two category variables are controlled: *AH*_*jk*_ represents human capital, including education, professional experience, the proportion of female employees, and the proportion of professionals and technicians, and *AX*_*jk*_ refers to the administrative-monopoly-related variables, such as the proportion of public employees, the proportion of state-owned capital, and the proportion of organizations with more than 500 employees.

As the administrative monopoly influences the union density, the administrative monopoly variables were used as instrumental variables for regression analysis in the second stage. Administrative-monopoly industries are typically composed of state-owned enterprises and/or government agencies. Although the reform changed the union membership structure to a certain extent, workers who remained in traditional state-owned industries have nearly never been outside unions. Following the start of the sectoral reform, the development of nonpublic sectors was strong. However, private capital rarely entered crucial industries with administrative monopolies; thus, these industries maintained a fairly high rate of union density. Considering the aforementioned conditions, administrative monopolies could well be correlated with union density. Therefore, this study took three variables of administrative monopolies (i.e., the ratio of public-sector capital, the ratio of state-owned employees, and the ratio of organizations with more than 500 employees) as instrumental variables (IVs) to regress union density. These were combined with safeguarding rights (SG), which can affect union density (labor dispute cases/10,000 individuals; casualties/10,000 individuals) because unions representing laborers in industries with greater risks may offer more incentives to join.


(5) $$\begin{array}{l} U_{jk}\;=\;c_{1}\;+\text{ φ}AX_{jk}\;+\text{ ϕ}SG_{jk}\;+\;e_{jk}, \end{array}$$

(6) $$\begin{array}{l} \tau_{jk}\;=\;c_{2}\;+\text{ λ}U_{jk}\;+\text{ γ}AH_{jk}\;+\;\varepsilon_{jk}, \end{array}$$

where λ is the coefficient that can measure how union density influences interindustry wage differentials by sector.

## Data

### Data source

The microlevel data used to regress in the first stage (e.g., Equation 1) were obtained from the China Urban Household Survey (UHS), conducted by the Economic Survey Team of the National Bureau of Statistics of China. The Tsinghua–China Data Center provides data access to the public. The UHS is a comprehensive survey of urban and rural households for three years, that is, 2004, 2008, and 2013. It includes more than 40,000 detailed data from four provinces, namely, Guangdong, Liaoning, Sichuan, and Shanghai (Eastern, Northeast, Western, and Southern China, respectively). These data were used as the samples in this study. The statistics include age, professional experience, gender, education, occupation, industry, wage, family status, and ethnicity; however, union status is excluded.

The independent variables in the second stage included union density, human capital, and administrative monopoly. These were obtained from three data sources, which required a significant amount of careful calculation. As the data mentioned above were from four provincial organizations and 3 years in the first stage, all the variables in the second stage needed to correspond to the time and space of the first-stage regression, and the industry also needed to be recalculated. The data on union density were calculated from the China Union Statistical Yearbook, which includes the numbers of union members and industry employees in various industries. The China Economic Census Yearbook includes the level of education required for the return, the proportion of technical personnel, and the proportion of women. The statistical yearbooks of each administrative region contain relevant information on publicly owned organizations in various regions, including the ratio of publicly owned capital, the ratio of public employees, and the ratio of organizations with more than 500 employees.

The most challenging part of the data processing involved the presence of serious statistical calibration inconsistencies in the three-year data. In 2004, the UHS classified Chinese industries into 16 categories according to the Chinese Industry Classification and Codes for National Economic Activities (GB 4754-84). However, in 2008, the National Bureau of Statistics referred to the International Standard Industrial Classification of All Economic Activities, re-established industry standards, and divided Chinese industries into 20 categories. Since there were few samples of the last industry, ‘others’, this article does not take them into account. After the classification standards were meticulously compared, the industry data from 2008 to 2013 were summarized into 15 categories (Appendix Table A). In other words, all the data in this study were recalculated according to industry-classification adjustments, such as *X*_*jk*_. With 15 industries and three kinds of ownership, public sector, private sector, and collective economy, there were 45 industry-sector categories. At the same time, since the existence of industry-sector dummy variables means that the data must represent a given industry by sector, all the remaining regression variables, including *U*_*jk*_, *AH*_*jk*_, and *AX*_*jk*_, were rearranged according to previously organized standards. In addition, individuals' education and marital status in the UHS was reclassified and recalculated.

### Descriptive statistics

The characteristics based on the UHS 2004, 2008, and 2013 data from different categories after recalculation are listed in Appendix Tables B1–B3. The overall sample includes 40,995 individual samples. Appendix Table B1 presents the classification based on basic information. Owing to the rapid pace of development in China, the economy developed swiftly during the period of 2004–2013; thus, the overall wage level demonstrated a general upward trend (which was likely to have a fixed time effect), and males had a higher income than females in these 3 years. In terms of education, in 2004, the average number of years of schooling of Chinese men was slightly higher than that of women. However, by 2013, women surpassed men, with 12.55 years of schooling. This result may have been due to the improvement in gender inequality in China in recent years. However, the gap in professional experience between men and women widened. Preferential policies were implemented for ethnic minorities, who may be treated unfairly by the Han owing to their small number; thus, they were also included as control variables. In the survey, marital status included single, married, divorced, widowed, and others. In the present study, marital status is divided into two categories: having a partner and not having a partner.

**Figure 4:**
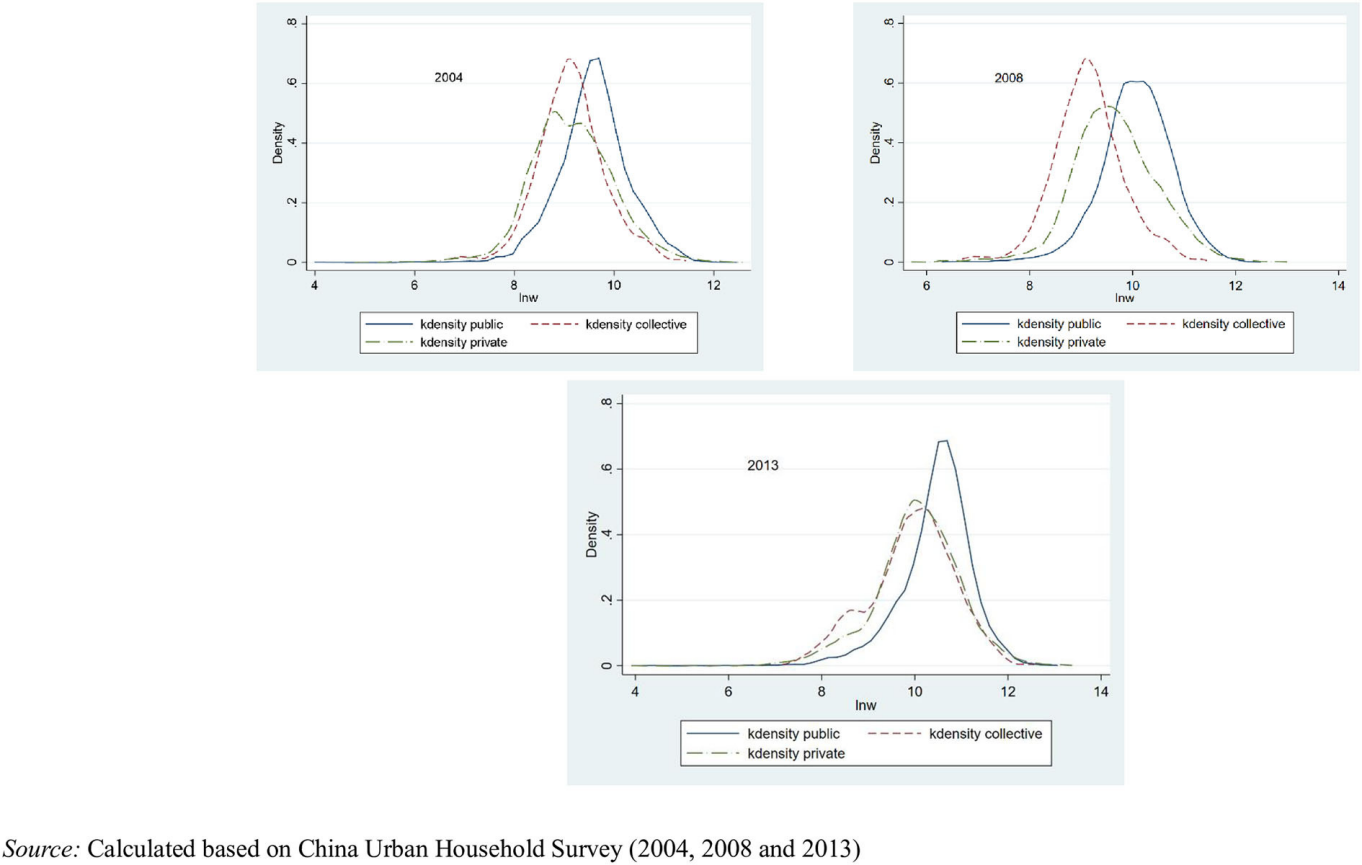
Kernel density distribution of wages by sector for 2004, 2008, and 2013.

Another key point is the employment distribution in different sectors (Appendix Table B2). Influenced by the former Soviet Union and socialist system, the early public sector accounted for the highest proportion of employees, reaching 55.27%. However, with the market reform and economic progress, this proportion dropped to 44.91%. By contrast, in the private sector, the number of employed individuals in 2013 exceeded half of all employed individuals, and this figure may continue to increase in the future. The collective economy has characteristics common to both the public and private sectors, which is another legacy of the former Soviet Union. Although the collective economy accounts for only ~5% of the total sample, it exerts some impact on wage differentials. Therefore, it is classified as a separate category in this study^1^. As mentioned above, the public sector generally consists of, or is related to, monopoly industries and is endorsed by the state or local governments; thus, income in the public sector is high. Figure 4 shows the Kernel density distribution of the logarithm of wages in 2004, 2008, and 2013.

In short, in the 3 years studied, three types of sector demonstrate a right-skewed trend, although wages were consistently high in the public sector. Moreover, the variance was small, which was consistent with expectations. Occupation was also a control variable. By comparing rough judgments, some service-related industries were developed, such as businesses, whereas the proportion of agriculture and industry-related occupations demonstrated a downward trend.

**Figure 5:**
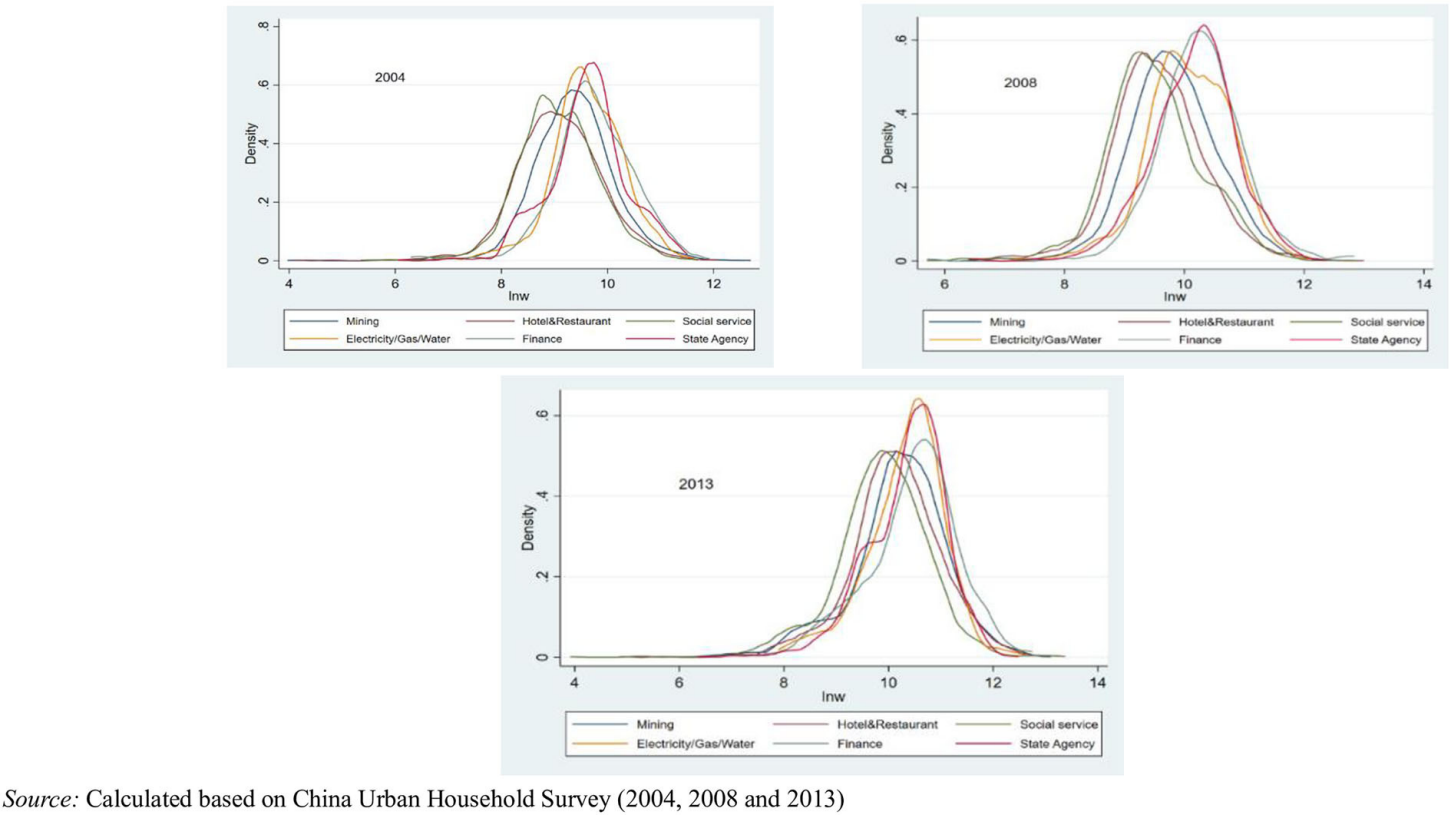
Kernel density distribution of wages by representative industry for 2004, 2008, and 2013.

Appendix Table B3 describes the proportion of employment based on industry classification. As the China Economic Census Yearbook has different statistical industry classifications for the 3 years, this study modified its classification scope, combined with the survey manual, and adopted the 15 categories for 2004 as the main categories. Few previous documents reclassify industry data and use them for statistical analysis; this is the main breakthrough and contribution of this study. From an overall point of view, the number of employed individuals was highest in the manufacturing, household and business service, hotel and restaurant, transportation, and information industries. Although the manufacturing industry accounted for the highest number of employed individuals, the proportion of the service industry increased rapidly. Figure 5 presents the Kernel density distribution of the logarithm of wages in 2004, 2008, and 2013 by industry. Although 15 categories are included, the figure depicts income levels in only six representative industries, including the mining, hotel and restaurant, and social service (which refers to household and business services in this study) industries. These private industries have a high degree of participation, making it difficult for monopolies to form. The latter industries in Figure 5, namely, finance and electricity, water, and gas, are often highly publicized in China; the last one is the government. Based on Figure 5, regardless of the year, income in the latter three categories was consistently the highest, and the wage differentials tended to widen.

To further describe how administrative monopolies influence union density in the public sector, the related data are presented in Appendix Table B4. The union density is high, and the industries are concentrated in electricity, gas, and water, financial intermediation, health, sports, and social welfare, and education, culture, and broadcasting, all of which feature a relatively high proportion of state-owned organizations. In the first two industries, monopolies easily form; they are essentially controlled by the state. The latter two industries have a high degree of government participation under the influence of socialist ideology. The proportion of state-owned-organization employment and the proportion of state-owned capital also behaved similarly. From the perspective of educational level, the education, science, technology, and finance industries had the highest academic requirements for qualification, whereas the mining industry had the lowest requirements. The proportion of technical personnel showed a similar pattern.

## Empirical analysis

### First-stage estimation and industry wages

Table 1 shows the explanatory power of the regressions when focusing the industry-sector dummies. As industry effects and covariates are not orthogonal to each other, the R^2^ of the first and second regressions did not add up to that of the third regression. The R^2^ of the industry-sector dummies was 13.1% for 2004 and fell slightly to 9.8% across the 3 years, which implies that industry affiliation can explain ~10% of individual wage dispersion. After individual and other occupation characteristics were controlled, as presented in columns (2) and (3), the explanatory power of the model increased, with the adjusted R^2^ ranging from 41.1% in 2004 to 31.5% in 2013. Through the comparison of the R^2^ in columns (2) and (3), the industry affiliation alone explained 4% and 3% of the wage variation in 2004 and 2013, respectively, which are lower than the specifications without controlling for individual and job-related characteristics. According to these results, industry–sector effects can explain ~4–13% of the wage variation.

**Table 1:** Explanatory power of wage equations (Dep. Variable: log yearly earnings).

	**2004**			**2008**			**2013**		
	**(1)**	**(2)**	**(3)**	**(1)**	**(2)**	**(3)**	**(1)**	**(2)**	**(3)**
Industry-Sector dummies	Yes	No	Yes	Yes	No	Yes	Yes	No	Yes
Control variable	No	Yes	Yes	No	Yes	Yes	No	Yes	Yes
N	12,603	12,603	12,603	13,920	13,920	13,920	14,461	14,461	14,461
adj. R^2^	0.402	0.131	0.441	0.293	0.115	0.311	0.282	0.098	0.315

The explanatory variables include years of schooling, working experience and its square, one marital status dummy (has partner or not), one ethnicity dummy (Han/non-Han), one gender dummy, six occupation dummies, three province dummies and forty-four (one digit) industry classification dummies.

Source: China Urban Household Survey (2004, 2008, and 2013).

Equation (1) was first regressed using the ordinary least squares (OLS) method, as heteroscedasticity can potentially influence the estimation. The WLS method can effectively control the impact of human capital factors, such as educational level, working experience and gender, and occupational factors on individual labor wages at the individual level, thereby effectively estimating the difference in labor wages caused by industry factors. Thus, the weighted least squares (WLS) method was applied for the regression, using the residuals as the weights.

The results are shown in Tables 2–4. Most of the coefficients of the control variables are significant. Education and professional experience positively affected wages, and men generally earned 19.1% more than women. The regression results for ethnicity were not significant, indicating that minorities were not discriminated against in the workplace in China. From the perspective of occupation, science-and-technology and public-administration managers earned the highest incomes, whereas agricultural workers earned the lowest incomes. Tables 2–4 also present the 44 coefficients of the industry–sector dummy variables. It is easily observed that the public sector enjoys a premium on wages, especially in monopolized industries.

**Table 2:** First-stage estimation for 2004.

**Control variable**		**Coff**	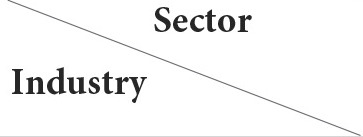	**Wage differentials coefficient (** ***α*** **)**		
				**Public sector**	**Collective economy**	**Private sector**
Exp	Working experience	0.0274^***^	Agriculture	0.232^**^	– 0.211^**^	0.370^***^
		(0.00221)		(0.101)	(0.0823)	(0.115)
Exp2	Working experience squared	– 0.000385^***^	Mining	0.444^***^	– 0.233	– 0.0194
		(0.0000498)		(0.0581)	(0.179)	(0.0844)
Edu	Education	0.0788^***^	Manufacturing	0.283^***^	0.0197	0.237^***^
		(0.00286)		(0.0516)	(0.0676)	(0.0526)
Gender	Male = 1	0.191^***^	Electricity, Gas and Water	0.490^***^	0.207	0.421^***^
		(0.0121)		(0.0641)	(0.133)	(0.102)
*Province*	Guangdong = 0		Construction	0.180^**^	0.183	0.220^***^
Liaoning	Located Northwest	0.732^***^		(0.0754)	(0.140)	(0.0633)
		(0.0190)	Water and Environment Management	0.417^***^	0.0846	0.171^*^
Shanghai	Located East	0.538^***^		(0.0947)	(0.209)	(0.103)
		(0.0154)	Transport and Information	0.491^***^	0.196^**^	0.345^***^
Sichuan	Located West	0.000401		(0.0572)	(0.0961)	(0.0532)
		(0.0150)	Wholesale and Retail, Hotel and Restaurants	0.105^*^	0.0158	0.130^**^
Marriage	Has Partner = 1	– 0.106^***^		(0.0594)	(0.0971)	(0.0514)
		(0.0204)	Financial Intermediation	0.336^***^	0.191	0.371^***^
Ethnicity	Han = 1	0.0192		(0.0629)	(0.119)	(0.0854)
		(0.0282)	Real Estate	0.190^**^	0.277^**^	0.158^**^
*Occupation*	Public Administration			(0.0824)	(0.132)	(0.0627)
	Manager = 0		Households and Business Services	0.217^***^	0.108	0.0800
Technician	Science & technology	0.0870^**^		(0.0582)	(0.0726)	(0.0509)
		(0.0364)	Health, sports and social welfare	0.337^***^	0.334^*^	0.169^**^
Clerk	Administrative & Business	– 0.0685^***^		(0.0630)	(0.199)	(0.0854)
		(0.0189)	Education, culture and broadcast	0.336^***^	0.312^**^	0.165^***^
Service	Household & Business	– 0.325^***^		(0.0568)	(0.129)	(0.0640)
		(0.0293)	Scientific Research	0.371^***^	– 0.141	0.482^***^
Agriculture	Agriculture Production	– 0.423^***^		(0.0750)	(0.202)	(0.121)
		(0.0234)	Social Organization	0.286^***^	0.240^**^	Base
Production	Production & Transport	– 0.304^***^			(0.121)	Group
		(0.0215)				
Soldier		– 0.618^***^				
		(0.159)				

*** *p* < 0.01,

** *p* < 0.05,

* *p* < 0.1.

Standard errors in parentheses.

**Table 3:** First-stage estimation for 2008.

**Control variable**		**Coff**.	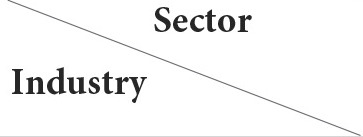	**Wage differentials coefficient (** ***α*** **)**		
				**Public sector**	**Collective economy**	**Private sector**
Exp	Working experience	0.0206^***^	Agriculture	0.241^**^	0.459^*^	0.108
		(0.00209)		(0.110)	(0.243)	(0.100)
Exp2	Working experience squared	– 0.000305^***^	Mining	0.662^***^	0.0871	0.348^***^
		(0.0000485)		(0.0803)	(0.212)	(0.115)
Edu	Education	0.0742^***^	Manufacturing	0.299^***^	0.108	0.166^**^
		(0.00266)		(0.0687)	(0.0844)	(0.0679)
Gender	Male = 1	0.204^***^	Electricity, Gas and Water	0.421^***^	0.376^*^	0.265^**^
		(0.0120)		(0.0764)	(0.193)	(0.104)
*Province*	Guangdong = 0		Construction	0.221^**^	0.0927	0.197^***^
Liaoning	Located Northwest	0.700^***^		(0.0922)	(0.136)	(0.0728)
		(0.0317)	Water and Environment Management	0.137	– 0.348	0.292^*^
Shanghai	Located East	0.402^***^		(0.0994)	(0.311)	(0.168)
		(0.0139)	Transport and Information	0.335^***^	0.186^*^	0.289^***^
Sichuan	Located West	0.700^***^		(0.0727)	(0.104)	(0.0691)
		(0.0117)	Wholesale and Retail, Hotel and Restaurants	0.0952	0.0649	0.136^**^
Marriage	Has Partner = 1	– 0.181^***^		(0.0845)	(0.0992)	(0.0674)
		(0.0196)	Financial Intermediation	0.444^***^	0.587^***^	0.406^***^
Ethnicity	Han = 1	– 0.0638^**^		(0.0802)	(0.126)	0.108
		(0.0319)	Real Estate	(0.0777)	0.415	0.321^***^
*Occupation*	Public Administration			0.127	(0.257)	(0.0838)
	Manager = 0		Households and Business Services	(0.128)	0.235^***^	0.0133
Technician	Science & technology	– 0.105^***^		0.0790	(0.0829)	(0.0670)
		(0.0353)	Health, sports and social welfare	(0.0793)	0.109	0.302^***^
Clerk	Administrative & Business	– 0.177^***^		0.307^***^	(0.158)	(0.0873)
		(0.0340)	Education, culture and broadcast	(0.0741)	0.455^***^	0.219^***^
Service	Household & Business	– 0.359^***^		0.208^***^	(0.132)	(0.0780)
		(0.0381)	Scientific Research	(0.0708)	0.0825	0.492^***^
Agriculture	Agriculture Production	– 0.382^***^		0.374^***^	(0.406)	(0.142)
		(0.0852)	Social Organization	(0.0872)	0.363^***^	Base
Production	Production & Transport	– 0.418^***^			(0.110)	Group
		(0.0374)				
Soldier		– 0.0557				
		(0.0931)				

*** *p* < 0.01,

** *p* < 0.05,

* *p* < 0.1.

Standard errors in parentheses.

**Table 4:** First-stage estimation for 2013.

**Control variable**		**Coff**.	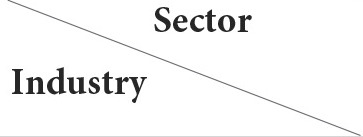	**Wage differentials coefficient (** ***α*** **)**		
				**Public sector**	**Collective economy**	**Private sector**
Exp	Working experience	0.0268^***^	Agriculture	0.271^**^	0.698^**^	0.157
		(0.00256)		(0.118)	(0.323)	(0.118)
Exp2	Working experience squared	– 0.000554^***^	Mining	0.685^***^	0.259	0.424^***^
		(0.0000552)		(0.0808)	(0.174)	(0.142)
Edu	Education	0.0905^***^	Manufacturing	0.383^***^	– 0.00863	0.154^***^
		(0.00311)		(0.0562)	(0.0983)	(0.0561)
Gender	Male = 1	0.232^***^	Electricity, Gas and Water	0.396^***^	– 0.140	0.281^***^
		(0.0141)		(0.0749)	(0.285)	(0.102)
*Province*	Guangdong = 0		Construction	0.371^***^	0.0181	0.252^***^
Liaoning	Located Northwest	0.599^***^		(0.0773)	(0.154)	(0.0642)
		(0.0229)	Water and Environment Management	0.310^***^	– 0.104	0.0789
Shanghai	Located East	– 0.152^***^		(0.109)	(0.245)	(0.156)
		(0.0180)	Transport and Information	0.414^***^	0.262^***^	0.271^***^
Sichuan	Located West	0.00470		(0.0619)	(0.0978)	(0.0548)
		(0.0171)	Wholesale and Retail, Hotel and Restaurants	0.234^***^	0.0798	0.340^***^
Marriage	Has Partner= 1	– 0.209^***^		(0.0765)	(0.138)	(0.0556)
		(0.0222)	Financial Intermediation	0.462^***^	0.0468	0.346^***^
Ethnicity	Han = 1	– 0.0242		(0.0738)	(0.169)	(0.0705)
		(0.0328)	Real Estate	0.174^*^	0.0878	0.302^***^
*Occupation*	Public Administration			(0.106)	(0.202)	(0.0721)
	Manager = 0		Households and Business Services	0.152^**^	– 0.0129	0.115^**^
Technician	Science & technology	– 0.279^***^		(0.0687)	(0.0904)	(0.0548)
		(0.0477)	Health, sports and social welfare	0.265^***^	– 0.292	0.165^*^
Clerk	Administrative & Business	– 0.408^***^		(0.0696)	(0.243)	(0.0888)
		(0.0466)	Education, culture and broadcast	0.288^***^	– 0.275^*^	0.0952
Service	Household & Business	– 0.454^***^		(0.0611)	(0.143)	(0.0705)
		(0.0497)	Scientific Research	0.438^***^	0.312^***^	0.122
Agriculture	Agriculture Production	– 0.827^***^		(0.0789)	(0.0557)	(0.148)
		(0.140)	Social Organization	0.274^***^	0.335^***^	Base
Production	Production & Transport	– 0.462^***^			(0.127)	Group
		(0.0503)				
Soldier		0.125				
		(0.130)				

*** *p* < 0.01,

** *p* < 0.05,

* *p* < 0.1.

Standard errors in parentheses.

Table 5 presents the adjusted interindustry wage differential coefficient according to Equations (2) and (3), which used wages in social organizations in the private sector as the base group. Overall, the wages in the electricity, gas, water, information and transportation, and finance industries were relatively high, whereas the income levels in the construction, manufacturing, and retail industries were relatively low.

**Table 5:** Adjusted wage differentials coefficient.

**Industry**	**2004**			**2008**			**2013**		
	**Public**	**Collective**	**Private**	**Public**	**Collective**	**Private**	**Public**	**Collective**	**Private**
Agriculture	– 0.0107207	– 0.4538518	0.1273958	0.031699	0.247451	– 0.10378	– 0.00827	0.418868	– 0.12266
Mining	0.2005657	– 0.4762975	– 0.262394	0.452869	– 0.12947	0.134453	0.405388	– 0.02088	0.144421
Manufacturing	0.0399177	– 0.2232639	– 0.006441	0.083564	– 0.10799	– 0.04955	0.103903	– 0.28819	– 0.1251
Electricity, Gas and Water	0.2471613	– 0.0357001	0.1775925	0.210664	0.162556	0.054095	0.116644	– 0.41911	0.001553
Construction	– 0.0625593	– 0.0601401	– 0.022843	0.01293	– 0.12139	– 0.01467	0.091615	– 0.26146	– 0.02752
Water and Environment Management	0.1737171	– 0.1583771	– 0.071917	– 0.07404	– 0.5649	0.079526	0.030377	– 0.38381	– 0.20064
Transport and Information	0.2475987	– 0.047439	0.1015612	0.122845	– 0.02915	0.076639	0.134781	– 0.01735	– 0.00833
Wholesale and Retail, Hotel and Restaurants	– 0.1375716	– 0.2271385	– 0.113146	– 0.11502	– 0.14204	– 0.07366	– 0.04531	– 0.19979	0.060005
Financial Intermediation	0.0933456	– 0.0522514	0.1284244	0.23588	0.378415	0.196295	0.182877	– 0.23275	0.066525
Real Estate	– 0.0527005	0.0336497	– 0.084996	– 0.08066	0.205793	0.108913	– 0.10544	– 0.19178	0.022304
Households and Business Services	– 0.0259292	– 0.1352849	– 0.162981	– 0.12971	0.02639	– 0.19882	– 0.128	– 0.29249	– 0.1641
Health, sports and social welfare	0.094006	0.0914999	– 0.073632	0.098756	– 0.1013	0.094489	– 0.01488	– 0.5717	– 0.11408
Education, Culture and Broadcast	0.0925789	0.0685644	– 0.077657	– 0.00073	0.242967	0.008021	0.007949	– 0.55419	– 0.18434
Scientific Research	0.1281857	– 0.3839293	0.2392014	0.165687	– 0.13147	0.279847	0.158081	– 0.19918	0.15723
Social Organization	0.0428859	– 0.0030403	–	0.092838	0.156204	–	– 0.00595	0.05516	–

Weights are included in all regressions, social organization in private sector is used as base group.

The wage differentials in the public sector and the other two sectors were huge. The income from mining in the public-sector system was much higher than that in the two other sector systems, because the state and local governments often control important mineral resources, whereas private individuals and collectives can only mine ordinary resources, such as coal. In the other industries prone to forming monopolies, such as water, electricity and gas, and transportation, their coefficients related to infrastructure construction were positive and had large absolute values. Education, social security, and scientific research in China are dominated mainly by central and local government, and occupy a certain share of the national fiscal budget; thus, their coefficient was ~10%. Most of the industries mentioned above are heavily controlled by the state and influenced by the Soviet Union and socialism (focusing on welfare and labor); thus, union density was consistently high. Wages in the private sector were at a disadvantage compared with those in the public sector in most industries. However, as entrepreneurs and managers are sensitive to the market, they pay considerable attention to innovation and technological progress. Moreover, they are engaged in wholesale and retail and finance, in which workers tend to earn high incomes. The performance of the coefficient of science and technology was excellent, reaching 23.9, 28.0, and 15.7% over the 3 years.

### Second-stage estimation: Wage differentials, union density, and administrative monopolies

In the second stage, the administrative monopolies and labor safeguards were used to perform a regression to union density, as in Equations (5) and (6).

First, we performed an exogeneity test on the overidentification of these IVs, as shown in Appendix Table C1. On the test, p was equal to 0.3394, which means the IVs were exogenous and successfully passed the exogeneity test. Further, we gave the F-statistics a value of 10.62 in the regression Appendix Table C2 to increase the effectiveness of the IVs.

The results of the first step of the IVs are presented in Appendix Table C3. Most of the coefficients were significant. According to the regression results of the first-step regression of the union density using the IV method, the regression coefficients of the public sector were 0.78, 0.336, and 0.355, which were all significant at the 1% level and exactly matched the idea that the public sector could lead to high union density. The number of companies with more than 500 employees as the proportion of the total number of companies in an industry also significantly affected the union density, because large companies are often controlled by the state and are monopolistic by nature. In addition, the regression results for the proportion of employed casualties were negative and significant at the 1% level, which indicated that union density was low in industries with a high degree of work-related risks. By strengthening the power of labor unions to safeguard rights, in the specific situation, the industries with the highest proportion of casualties mainly included mining, construction, wholesale and retail, and manufacturing. The union density in these industries was lower than that in the other industries with an administrative-monopoly nature.

The OLS and IV results are presented in Table 6. The coefficients of the OLS were higher than those in the IV as they did not consider the influence of administrative monopolies and labor safeguards, which caused the upward biased estimation. The coefficients of the unions in the 3 years were 0.714, 0.497, and 0.0933 and significant at least at the 10% level, which meant that the unions had a significant impact on the wage differentials. However, the influence of union density is weakening, which is in line with the basic perception of China's economic development; that is, at the beginning of the market reforms, most industries were in public sector, meaning that unions could rely on state power and influence, which were relatively strong. Nevertheless, the current public sector (especially state-owned and local enterprises) is declining, whereas the economy of the private sector is developing rapidly. Unions in the private sector are immature and cannot organize effective negotiations or obtain strong bargaining power. Rather, from the perspective of human capital, wages are closely linked to educational level. Moreover, increasing the proportion of women in employment can also reduce wage differentials in industries in the future.

**Table 6:** OLS and IV estimation: Union density and wage differentials.

	**2004**		**2008**		**2013**	
	**OLS**	**IV**	**OLS**	**IV**	**OLS**	**IV**
Union density	0.714^*^	0.536^**^	0.497^**^	0.421^**^	0.283	0.0933^*^
	(0.384)	(0.257)	(0.187)	(0.199)	(0.228)	(0.050)
Education	0.0610	0.0833^**^	0.0745^**^	0.0708^**^	0.0278	0.0230^**^
	(0.0444)	(0.0413)	(0.0301)	(0.0286)	(0.0304)	(0.009)
Technician ratio	– 0.180	– 0.341	– 0.0475	– 0.0817	– 0.358	– 0.237
	(0.258)	(0.280)	(0.221)	(0.211)	(0.258)	(0.307)
Female ratio	– 0.112	– 0.0648^*^	– 0.428^*^	– 0.394^*^	– 0.490	– 0.558^*^
	(0.265)	(0.038)	(0.246)	(0.234)	(0.341)	(0.326)
Public sector capital ratio	– 0.328		– 0.178		– 0.0755	
	(0.323)		(0.116)		(0.124)	
Public sector	– 0.334		– 0.027		– 0.00733	
employee ratio	(0.223)		(0.064)		(0.169)	
Above 500 people	– 2.069		– 0.0918		0.561	
organization ratio	(1.485)		(0.152)		(0.766)	
_cons	– 0.927^*^	– 1.138^**^	– 0.938^**^	– 0.898^***^	– 0.356	– 0.217
	(0.513)	(0.496)	(0.363)	(0.343)	(0.340)	(0.354)
*N*	44	44	44	44	44	44
adj. *R*^2^	0.070	0.120	0.170	0.158	0.099	0.078

* *p* < 0.1,

** *p* < 0.05,

*** *p* < 0.01,

using public-sector capital ratio, public-owned employee ratio and above 500 people organization ratio, labor dispute case/10000 person, casualties/10000 person as IV. Standard errors in parentheses.

## Conclusion and policy suggestions

### Results discussion

Using more than 40,000 individual data from 3 years of UHS, this article discusses how union density influences the interindustry wage differentials by sector in China by using the two-stage method, in which administrative monopolies are considered as IVs.

**Figure 6:**
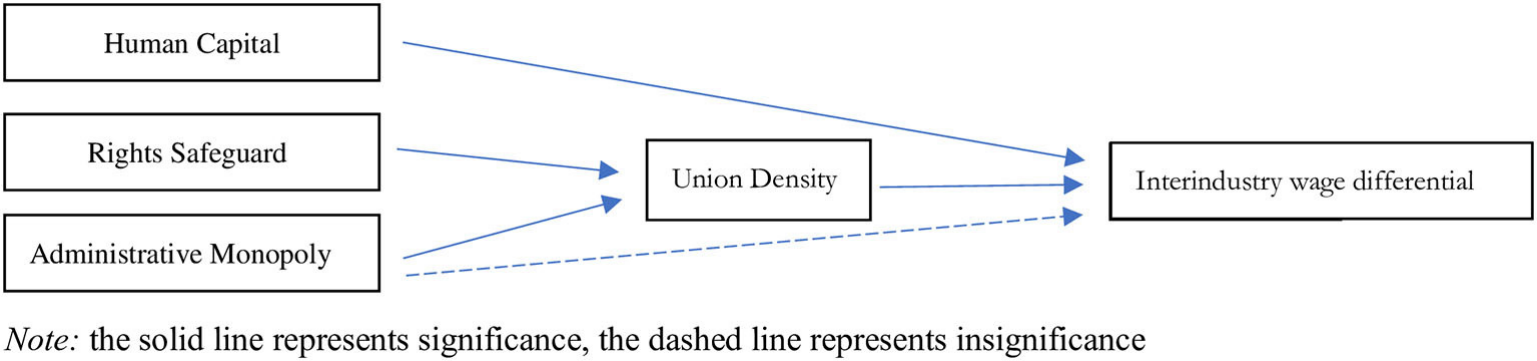
The relationship between union density and wage.

According to the results of the second stage, the influence of union density can increase the interindustry wage differentials by sector, although this trend is declining, as unions are growing in the private sector, where their bargaining power is weak, which proves that Hypothesis 1 is correct. The results are similar to those of Ge (2007), Anwar and Sun (2012), and Zhang et al. (2011). The IV regression results in the second stage demonstrated that the administration can strongly affect wage differentials indirectly, through union density, instead of directly, which contradicts Hypothesis 2 and proves the correctness of Hypothesis 3. These results are similar to those of Zhang et al. (2011). This paper further found that union density is an indirect way for government and CCP to influence wage differentials. Furthermore, Hypothesis 4 was proven to be true, as human capital can influence interindustry wage differentials directly. The framework of these four Hypotheses is drawn in Figure 6.

This conclusion reveals the underlying effects of China's market reformation on its wages and economy. Although the density of Chinese unions and their member numbers have increased steadily in recent years, numerous industries must co-exist in administrative and nonadministrative monopolies. The unions and workers in state-owned enterprises are affected by administrative monopolies, with higher incomes and greater labor safeguards. However, in the private sector and collective economy, owing to their short establishment period and lack of experienced unions, as well as the inaccessibility of certain industries, the compensation for workers is relatively low.

### Policy suggestions

The key to eliminating interindustry wage differentials is to decrease the influence of administration on union density. First, union work could focus on supporting grassroots unions in nonadministrative monopolies, as grassroots unions are more independent and be influenced less by administrative monopolies. Second, against the background of the gradual advancement of administrative monopoly reforms, some administrative monopoly industries should open further to market competition and the private sector. Moreover, private capital and management methods should gradually be employed in certain administrative monopoly industry chains, which will also lead to a wider income distribution. Third, the relevant labor-management relations will become increasingly complex in the future, and the demand for economic rights and protections for workers from unions will increase correspondingly. In short, in response to emerging wage-distribution problems, private nonmonopoly industries should enhance unions' ability to establish an effective wage-distribution-coordination mechanism.

### Future directions

As the data from the UHS and Yearbook are not continuous in years and feature different individual samples, panel data for further in-depth regression analyses are necessary. Furthermore, the control variables selected for the second-stage regression to represent the administration may be insufficient. Furthermore, this paper does not consider the heterogeneity of representative industries enough to supplement the current study. Finally, the relation between union operating modes and interindustry wage differentials in China is worthy of further exploration.

## Data availability statement

The original contributions presented in the study are included in the article/Supplementary material, further inquiries can be directed to the corresponding author/s.

## Author contributions

ML: conceptualization, data curation, formal analysis, investigation, methodology, resources, visualization, writing-review and editing, and approved the submitted version.

## Conflict of interest

The author declares that the research was conducted in the absence of any commercial or financial relationships that could be construed as a potential conflict of interest. The reviewer AS-M declared a past collaboration with the author ML.

## Publisher's note

All claims expressed in this article are solely those of the authors and do not necessarily represent those of their affiliated organizations, or those of the publisher, the editors and the reviewers. Any product that may be evaluated in this article, or claim that may be made by its manufacturer, is not guaranteed or endorsed by the publisher.

## Footnotes

^1^The collective economy is a publicly owned economy in which the means of production are collectively owned by some workers.
