# Supplementary material for: The interindustry wage differentials by sector in China: What is the role of union density?
Source: Front Sociol. 2022 Sep 23;7:949293. doi: 10.3389/fsoc.2022.949293 (PMC9539559; doi:10.3389/fsoc.2022.949293)
Supplement: Supplementary file 1 [file Table_1.docx]

**Appendix**

**Table A Industry-Classification in 2004, 2008 and 2013**

| 15-category Industry (2004) | 20-Category Industry (2008/2013) |
| --- | --- |
| Agriculture | Agriculture, forestry, animal husbandry and fishery |
| Mining | Mining |
| Manufacturing | Manufacturing |
| Electricity, Gas and Water | Electricity, gas and water |
| Construction | Construction |
| Water and Environment Management | Transportation, warehousing and postal industry |
| Transport and Information | Information transmission, computer service and software |
| Wholesale and Retail, Hotel and Restaurants | Wholesale and retail |
| Financial Intermediation | Accommodation and Catering |
| Real Estate | Financial |
| Households and Business Services | Real estate |
| Health, sports and social welfare | Leasing and business services |
| Education, culture and broadcast | Scientific research, technical services and geological prospecting industry |
| Scientific Research | Water conservancy, environment and public facilities management |
| State and social organization | Resident services and other services; Education; Health, Social welfare; Culture, sports and entertainment; Public Administration and Social  Organization |
|  |  |
|  |  |

*Source:* China Urban Household Survey (2004, 2008 and 2013)

**Table B1 Descriptive Statistics of Basic Information**

|  | 2004 | | |  | 2008 | | |  | 2013 | | |  | All | | |
| --- | --- | --- | --- | --- | --- | --- | --- | --- | --- | --- | --- | --- | --- | --- | --- |
|  | Male | Female | Total |  | Male | Female | Total |  | Male | Female | Total |  | Male | Female | Total |
| Wage | 18382.45 | 13549.11 | 16225.23 |  | 28150.95 | 21793.81 | 25392.48 |  | 42609.69 | 32290.2 | 38054.02 |  | 30268.11 | 22934.81 | 27038.56 |
|  | (16584.67) | (12477.53) | (15084.24) |  | (24613.64) | (20556.77) | (23156.15) |  | (39681.95) | (31556.75) | (36678.32) |  | (30730.25) | (24512.59) | (28395.70) |
| Edu | 11.903 | 11.848 | 11.878 |  | 12.083 | 12.112 | 12.096 |  | 12.473 | 12.552 | 12.508 |  | 12.165 | 12.185 | 12.174 |
|  | (2.52) | (2.29) | (2.42) |  | (2.65) | (2.57) | (2.61) |  | (2.67) | (2.59) | (2.63) |  | (2.63) | (2.51) | (2.58) |
| Exp | 22.338 | 19.065 | 20.877 |  | 21.190 | 17.608 | 19.636 |  | 23.026 | 19.173 | 21.325 |  | 22.186 | 18.615 | 20.613 |
|  | (10.68) | (9.95) | (10.48) |  | (10.67) | (9.52) | (10.34) |  | (11.24) | (10.10) | (10.92) |  | (10.90) | (9.89) | (10.62) |
| Ethnicity | 0.038 | 0.046 | 0.041 |  | 0.031 | 0.035 | 0.033 |  | 0.043 | 0.046 | 0.044 |  | 0.037 | 0.042 | 0.039 |
| （0=Han 1=Others） | (0.19) | (0.21) | (0.20) |  | (0.17) | (0.18) | (0.18) |  | (0.20) | (0.21) | (0.21) |  | (0.19) | (0.20) | (0.19) |
| Marriage | 0.885 | 0.860 | 0.873 |  | 0.892 | 0.869 | 0.88 |  | 0.884 | 0.847 | 0.868 |  | 0.886 | 0.858 | 0.874 |
| （1= Has partner） | (0.32) | (0.35) | (0.33) |  | (0.31) | (0.34) | (0.32) |  | (0.32) | (0.36) | (0.34) |  | (0.32) | (0.35) | (0.33) |
| Sample Number | 6,983 | 5,629 | 12,612 |  | 7,881 | 6,041 | 13,922 |  | 8,077 | 6,384 | 14,461 |  | 22,941 | 18,054 | 40,995 |

*Note:* (1) In the UHS database, the degree of education is divided into eight categories: postgraduate, university, junior college, technical secondary school, high school, junior high school, elementary school, and illiterate. Because the number of illiterate people is too small, in actual research, it is classified as elementary school. According to China's education system, the corresponding education years are 18, 16, 14, 12, 12, 9, and 6. (2) Marital status includes the following: unmarried, married, divorced, widowed, and others. According to statistical needs, it is divided into whether there is a partner

*Source:* China Urban Household Survey (2004, 2008 and 2013)

**Table B2 Descriptive Statistics by Sector and by Occupation**

|  | 2004 | | |  | 2008 | | |  | 2013 | | |  | All | | |
| --- | --- | --- | --- | --- | --- | --- | --- | --- | --- | --- | --- | --- | --- | --- | --- |
|  | Male | Female | Total |  | Male | Female | Total |  | Male | Female | Total |  | Male | Female | Total |
| Sector (%) |  |  |  |  |  |  |  |  |  |  |  |  |  |  |  |
| Public Sector | 61.49 | 47.56 | 55.27 |  | 44.96 | 37.36 | 41.66 |  | 42.87 | 34.07 | 38.99 |  | 49.26 | 39.38 | 44.91 |
| Private Sector | 33.09 | 43.95 | 37.94 |  | 50.65 | 57.71 | 53.71 |  | 54.33 | 62.73 | 58.04 |  | 46.60 | 55.20 | 50.39 |
| Collective Economy | 5.41 | 8.49 | 6.79 |  | 4.39 | 4.93 | 4.63 |  | 2.80 | 3.20 | 2.97 |  | 4.14 | 5.43 | 4.71 |
| Occ (%) |  |  |  |  |  |  |  |  |  |  |  |  |  |  |  |
| Public Sector Manager | 4.78 | 1.40 | 3.27 |  | 5.02 | 1.92 | 3.68 |  | 3.34 | 1.08 | 2.34 |  | 4.36 | 1.46 | 3.08 |
| Technique & Research | 17.11 | 16.26 | 16.73 |  | 22.75 | 19.27 | 21.24 |  | 23.70 | 18.45 | 21.38 |  | 21.37 | 18.04 | 19.90 |
| Clerks | 27.58 | 29.40 | 28.39 |  | 25.50 | 29.65 | 27.30 |  | 30.25 | 35.98 | 32.78 |  | 27.81 | 31.81 | 29.57 |
| Household & Business Service | 6.29 | 11.74 | 8.72 |  | 17.89 | 31.68 | 23.88 |  | 17.59 | 31.97 | 23.94 |  | 14.25 | 25.57 | 19.24 |
| Agriculture | 10.60 | 24.11 | 16.63 |  | 0.62 | 0.41 | 0.53 |  | 0.35 | 0.19 | 0.28 |  | 3.56 | 7.72 | 5.39 |
| Production & Transport | 31.62 | 14.90 | 24.16 |  | 21.10 | 9.75 | 16.18 |  | 19.49 | 6.42 | 13.72 |  | 18.17 | 5.56 | 15.38 |
| Soldier | 0.20 | 0.09 | 0.50 |  | 0.69 | 0.18 | 0.47 |  | 0.61 | 0.05 | 0.36 |  | 0..07 | 4.72 | 7.72 |
| Others | 1.82 | 2.10 | 1.94 |  | 6.42 | 7.13 | 6.73 |  | 4.68 | 5.86 | 5.20 |  | 4.41 | 5.11 | 4.72 |
| Sample Number | 6,983 | 5,629 | 12,612 |  | 7,881 | 6,041 | 13,922 |  | 8,077 | 6,384 | 14,461 |  | 22,941 | 18,054 | 40,995 |

*Note:* (1) Public Sector refers to working in party and government agencies, state-owned enterprises, and institutions at all levels (2) Private Sector refers to working in private-owned organizations (3) Collective economy refers to working in urban collective firms and their management departments

*Source:* China Urban Household Survey (2004, 2008 and 2013)

**Table B3 Descriptive Statistics by Industry**

|  | 2004 | | |  | 2008 | | |  | 2013 | | |  | All | | |
| --- | --- | --- | --- | --- | --- | --- | --- | --- | --- | --- | --- | --- | --- | --- | --- |
|  | Male | Female | Total |  | Male | Female | Total |  | Male | Female | Total |  | Male | Female | Total |
| Industry (%) |  |  |  |  |  |  |  |  |  |  |  |  |  |  |  |
| Agriculture | 1.02 | 0.71 | 0.88 |  | 1.10 | 0.61 | 0.89 |  | 1.00 | 0.63 | 0.84 |  | 1.04 | 0.65 | 0.87 |
| Mining | 1.85 | 0.55 | 1.27 |  | 2.26 | 1.22 | 1.81 |  | 2.13 | 0.66 | 1.48 |  | 2.09 | 0.81 | 1.53 |
| Manufacturing | 25.73 | 16.97 | 21.82 |  | 20.34 | 13.62 | 17.43 |  | 19.75 | 12.08 | 16.36 |  | 21.77 | 14.12 | 18.40 |
| Electricity, Gas and Water | 3.85 | 2.04 | 3.04 |  | 3.68 | 2.07 | 2.98 |  | 3.23 | 1.43 | 2.43 |  | 3.57 | 1.83 | 2.81 |
| Construction | 4.01 | 1.51 | 2.89 |  | 4.62 | 1.85 | 3.42 |  | 5.42 | 1.94 | 3.89 |  | 4.72 | 1.78 | 3.42 |
| Water and Environment Management | 1.40 | 1.10 | 1.27 |  | 1.22 | 0.81 | 1.04 |  | 0.99 | 0.81 | 0.91 |  | 1.19 | 0.90 | 1.07 |
| Transport and Information | 13.42 | 5.35 | 9.82 |  | 13.44 | 5.69 | 10.08 |  | 20.29 | 19.19 | 19.80 |  | 15.84 | 10.36 | 13.43 |
| Wholesale and Retail, Hotel and Restaurants | 13.05 | 19.51 | 15.93 |  | 13.70 | 23.69 | 18.04 |  | 8.42 | 10.78 | 9.46 |  | 11.64 | 17.82 | 14.36 |
| Financial Intermediation | 2.49 | 2.47 | 2.48 |  | 2.84 | 3.58 | 3.16 |  | 3.39 | 3.99 | 3.66 |  | 2.93 | 3.38 | 3.13 |
| Real Estate | 2.62 | 3.04 | 2.81 |  | 1.46 | 1.03 | 1.27 |  | 2.08 | 1.69 | 1.91 |  | 2.03 | 1.89 | 1.97 |
| Households and Business Services | 8.52 | 19.12 | 13.25 |  | 13.23 | 19.48 | 15.95 |  | 10.75 | 18.80 | 14.30 |  | 10.92 | 19.13 | 14.54 |
| Health, sports and social welfare | 2.06 | 4.51 | 3.16 |  | 2.72 | 5.99 | 4.14 |  | 2.67 | 5.34 | 3.85 |  | 2.50 | 5.30 | 3.73 |
| Education, culture and broadcast | 6.30 | 8.78 | 7.41 |  | 5.79 | 9.09 | 7.22 |  | 5.39 | 9.21 | 7.07 |  | 5.80 | 9.03 | 7.23 |
| Scientific Research | 2.23 | 1.78 | 2.03 |  | 1.48 | 0.94 | 1.25 |  | 1.66 | 0.88 | 1.31 |  | 1.77 | 1.18 | 1.51 |
| Social Organization | 11.31 | 12.58 | 11.88 |  | 12.12 | 10.28 | 11.32 |  | 12.83 | 12.58 | 12.72 |  | 12.12 | 11.81 | 11.98 |
| Sample Number | 6,983 | 5,629 | 12,612 |  | 7,881 | 6,041 | 13,922 |  | 8,077 | 6,384 | 14,461 |  | 22,941 | 18,054 | 40,995 |

*Note:* The UHS's statistical division of industries has changed in the past three years. This article re-divides the latest 20 industries into the original 15 industries

*Source:* China Urban Household Survey (2004, 2008 and 2013)

**Table B4 Descriptive Statistics of Industrial Characteristics in Public-Sector**

| Industry | Union  Density | Public-Owned  Employee | Public-Owned  Capital | Average  education | Employees  above 500 | Technician | Female |
| --- | --- | --- | --- | --- | --- | --- | --- |
| 2004 |  |  |  |  |  |  |  |
| Agriculture | 0.959 | 0.364 | 0.819 | 10.404 | 0.177 | 0.168 | 0.335 |
| Mining | 0.699 | 0.579 | 0.268 | 10.239 | 0.019 | 0.115 | 0.165 |
| Manufacturing | 0.498 | 0.222 | 0.114 | 10.585 | 0.023 | 0.081 | 0.480 |
| Electricity, Gas and Water | 0.880 | 0.639 | 0.431 | 11.666 | 0.024 | 0.235 | 0.301 |
| Construction | 0.606 | 0.291 | 0.299 | 10.642 | 0.070 | 0.178 | 0.127 |
| Water and Environment Management | 0.738 | 0.755 | 0.125 | 10.980 | 0.006 | 0.130 | 0.394 |
| Transport and Information | 0.711 | 0.631 | 0.443 | 11.640 | 0.013 | 0.154 | 0.286 |
| Wholesale and Retail, Hotel  and Restaurants | 0.411 | 0.304 | 0.361 | 11.778 | 0.002 | 0.103 | 0.460 |
| Financial Intermediation | 0.862 | 0.573 | 0.420 | 13.571 | 0.073 | 0.408 | 0.505 |
| Real Estate | 0.447 | 0.236 | 0.124 | 11.492 | 0.006 | 0.163 | 0.385 |
| Households and Business Services | 0.375 | 0.398 | 0.170 | 11.988 | 0.003 | 0.137 | 0.369 |
| Health, Sports and Social welfare | 0.798 | 0.719 | 0.411 | 12.644 | 0.004 | 0.696 | 0.599 |
| Education, Culture and Broadcast | 0.827 | 0.852 | 0.303 | 13.668 | 0.003 | 0.688 | 0.511 |
| Scientific Research | 0.622 | 0.707 | 0.225 | 13.490 | 0.003 | 0.462 | 0.294 |
| Social Organization | 0.570 | 0.894 | 0.587 | 12.942 | 0.007 | 0.115 | 0.268 |
| 2008 |  |  |  |  |  |  |  |
| Agriculture | 0.921 | 0.950 | 0.595 | 10.668 | 0.198 | 0.102 | 0.322 |
| Mining | 0.659 | 0.601 | 0.431 | 10.701 | 0.020 | 0.121 | 0.156 |
| Manufacturing | 0.441 | 0.193 | 0.104 | 10.916 | 0.025 | 0.071 | 0.425 |
| Electricity, Gas and Water | 0.781 | 0.601 | 0.456 | 11.946 | 0.013 | 0.219 | 0.267 |
| Construction | 0.545 | 0.274 | 0.239 | 10.850 | 0.055 | 0.158 | 0.114 |
| Water and Environment Management | 0.715 | 0.886 | 0.355 | 11.390 | 0.011 | 0.120 | 0.382 |
| Transport and Information | 0.711 | 0.575 | 0.363 | 12.464 | 0.012 | 0.116 | 0.286 |
| Wholesale and Retail, Hotel  and Restaurants | 0.399 | 0.189 | 0.146 | 12.084 | 0.002 | 0.079 | 0.446 |
| Financial Intermediation | 0.863 | 0.386 | 0.350 | 14.076 | 0.105 | 0.284 | 0.505 |
| Real Estate | 0.376 | 0.265 | 0.053 | 12.110 | 0.006 | 0.146 | 0.323 |
| Households and Business Services | 0.381 | 0.313 | 0.254 | 12.302 | 0.004 | 0.101 | 0.374 |
| Health, sports and social welfare | 0.794 | 0.859 | 0.150 | 13.262 | 0.005 | 0.647 | 0.595 |
| Education, Culture and Broadcast | 0.828 | 0.967 | 0.377 | 14.143 | 0.004 | 0.614 | 0.512 |
| Scientific Research | 0.636 | 0.879 | 0.275 | 13.937 | 0.003 | 0.383 | 0.301 |
| Social Organization | 0.587 | 0.998 | 0.573 | 13.297 | 0.013 | 0.100 | 0.276 |
| 2013 |  |  |  |  |  |  |  |
| Agriculture | 0.783 | 0.968 | 0.184 | 12.923 | 0.183 | 0.074 | 0.274 |
| Mining | 0.616 | 0.193 | 0.007 | 11.049 | 0.024 | 0.140 | 0.145 |
| Manufacturing | 0.414 | 0.070 | 0.012 | 11.668 | 0.049 | 0.063 | 0.399 |
| Electricity, Gas and Water | 0.746 | 0.527 | 0.104 | 12.806 | 0.004 | 0.206 | 0.255 |
| Construction | 0.539 | 0.133 | 0.101 | 11.234 | 0.053 | 0.142 | 0.113 |
| Water and Environment Management | 0.731 | 0.799 | 0.216 | 12.540 | 0.027 | 0.114 | 0.390 |
| Transport and Information | 0.723 | 0.229 | 0.122 | 14.183 | 0.013 | 0.088 | 0.291 |
| Wholesale and Retail, Hotel  and Restaurants | 0.400 | 0.127 | 0.030 | 12.863 | 0.005 | 0.060 | 0.447 |
| Financial Intermediation | 0.850 | 0.323 | 0.231 | 16.050 | 0.151 | 0.202 | 0.498 |
| Real Estate | 0.406 | 0.101 | 0.037 | 13.642 | 0.020 | 0.128 | 0.349 |
| Households and Business Services | 0.378 | 0.227 | 0.055 | 12.825 | 0.009 | 0.073 | 0.372 |
| Health, sports and Social welfare | 0.821 | 0.873 | 0.191 | 15.610 | 0.003 | 0.607 | 0.616 |
| Education, Culture and Broadcast | 0.887 | 0.926 | 0.082 | 16.148 | 0.005 | 0.565 | 0.548 |
| Scientific Research | 0.660 | 0.588 | 0.102 | 14.992 | 0.004 | 0.316 | 0.312 |
| Social Organization | 0.647 | 0.994 | 0.612 | 14.502 | 0.031 | 0.090 | 0.305 |

*Note:* (1) Except for education, all other variables are ratios (2) Due to the limitation of the layout, this article only shows the characteristics of the industry under the condition of public sector

*Source:* China Statistical Yearbook by province, China Labor Statistical Yearbook (2004, 2008 and 2013)

**Table C1 Test of overidentifying restrictions**

Score chi2(1) = .912639 (p = 0.3394)

**Table C2 F statistics in First stage**

| Variable | R-sq. | Adjusted | Partial | Robust | Prob > F |  |
| --- | --- | --- | --- | --- | --- | --- |
|  |  |  |  |  |  |  |
| Union Density | 0.9307 | 0.9149 | 0.5097 | 10.6263 | 0.0002 |  |

**Table C3 First Step of IV for Administration Monopoly and Union Density**

|  | 2004 | 2008 | 2013 |
| --- | --- | --- | --- |
| Public sector employee ratio | 0.780*** | 0.336*** | 0.355*** |
|  | (0.0599) | (0.0667) | (0.0429) |
|  |  |  |  |
| Public sector | -0.189** | -0.0965 | -0.346*** |
| capital ratio | (0.0843) | (0.130) | (0.0353) |
|  |  |  |  |
| Above 500 people | 3.455*** | 1.854*** | 1.545*** |
| organization ratio | (0.282) | (0.293) | (0.178) |
|  |  |  |  |
| Labor dispute case  /10000 person | 0.00820*** | 0.00359*** | 0.00178 |
|  | (0.00274) | (0.00108) | (0.00327) |
|  |  |  |  |
| Casualties | -0.00351 | -0.0326*** | -0.0173*** |
| /10000 person | (0.00265) | (0.00789) | (0.00487) |
|  |  |  |  |
| _cons | 0.133** | 0.468*** | 0.561*** |
|  | (0.0614) | (0.0428) | (0.0509) |
| N | 44 | 44 | 44 |
| adj. R2 | 0.812 | 0.683 | 0.715 |

*Note:* * p < 0.1, ** p < 0.05, *** p < 0.01 Standard errors in parentheses

*Source:* China Statistical Yearbook by province (2004, 2008 and 2013), China Labor Statistical Yearbook (2004, 2008 and 2013)
